# The effect of pathological fractures on the prognosis of patients with osteosarcoma: a meta-analysis of 14 studies

**DOI:** 10.18632/oncotarget.20375

**Published:** 2017-08-21

**Authors:** Yifei Zhou, Qian Lu, Jifeng Xu, Ruijian Yan, Junkun Zhu, Juntao Xu, Xuesheng Jiang, Jianyou Li, Fengfeng Wu

**Affiliations:** ^1^ Department of Orthopedics, The Second Affiliated Hospital and Yuying Children's Hospital of Wenzhou Medical University, Wenzhou, Zhejiang, China; ^2^ Department of Orthopedics, Huzhou Central Hospital, Zhejiang University Huzhou Hospital, Huzhou, Zhejiang, China; ^3^ Department of Orthopedics, Zhejiang Provincial People's Hospital, People's Hospital of Hangzhou Medical College, Hangzhou, Zhejiang, China; ^4^ Department of Orthopedics, The Second Affiliated Hospital of Medicine College, Hangzhou, Zhejiang University, Zhejiang, China; ^5^ Department of Orthopedics, Lishui Central Hospital, Lishui, Zhejiang, China; ^6^ Department of Orthopedics, Huzhou Hospital of Traditional Chinese Medicine, Huzhou, Zhejiang, China

**Keywords:** pathological fracture, osteosarcoma, meta-analysis

## Abstract

Osteosarcoma is a leading cause of malignant tumor related death. We conducted a meta-analysis to evaluate the association between pathological fractures and prognosis in patients with osteosarcoma. We searched PubMed, Web of Science, and Embase for studies published until May 15, 2017. Crude and adjusted relative risk (RR) with 95% confidence intervals were used to compare data between the case and control groups. Fourteen studies and 3910 patients were included in the final meta-analysis. No statistically significant difference was detected between the pathological fracture and non-pathological fracture groups in local recurrences analysis (RR = 1.102, 95% CI: 0.813–1.495, *P* = 0.531); however, a statistically significant difference was found between group in distant metastasis (RR = 1.424, 95% CI: 1.089–1.862, *P* = 0.01). For survival rates, the following RRs were calculated: 3-year overall survival (OS) (RR = 0.736, 95% CI: 0.593–0.912, *P* = 0.005); 5-year OS (RR = 0.889, 95% CI: 0.791–0.999, *P* = 0.049); 3-year event-free survival (EFS) (RR = 0.812, 95% CI: 0.682–0.966, *P* = 0.018); and 5-year EFS (RR = 0.876, 95% CI: 0.785–0.978, *P* = 0.019). The pooled estimate of RR was 0.673 (95% CI: 0.364–1.244, *P* = 0.206) for local recurrence in the amputation and limb salvage groups. In conclusion, our analysis indicated that there were no differences in local recurrence and local recurrence after limb salvage between patients with or without a fracture. Additionally, the patients with pathological fracture had a higher risk of distant metastasis and lower 3-year OS, 5-year OS, 3-year EFS, and 5-year EFS. Considering the limitations of this study, we believe that future large-scale studies should be performed to confirm our conclusions.

## INTRODUCTION

Osteosarcoma, the most common primary malignancy of bone, is a leading cause of malignant tumor related death in children and adolescents [1]. In the USA, nearly 850 new cases are diagnosed each year, and about 400 cases arise in children and adolescents ≤ 20 years old [2]. More than 20% of cases involve pulmonary metastasis when they are diagnosed as osteosarcoma, which frequently results in patient death [3]. Due to frequent lung metastasis, the prognosis for osteosarcoma is poor [4]. Currently, the usual treatment for osteosarcoma is complete radical, surgical, en bloc resection with adjuvant chemotherapy after operation or neoadjuvant chemotherapy before surgical resection of the primary tumor [5]. Despite combined therapy, more than 30% of patients show recurrence or metastatic disease in the first 5 years after diagnosis [6].

Pathological fractures have important prognostic and treatment implications for patients with osteosarcoma [7]. About 5%–10% of osteosarcoma patients have a pathological fracture [8]. Theoretically, pathological fracture can worsen prognosis of osteosarcoma due to fracture hematoma, or by aiding the spread of micro-metastases. Previously, Xie et al. [9] found that the risk of local recurrence or distal metastasis did not seem significantly increased in osteosarcoma patients with pathological fracture. However, osteosarcoma patients with pathological fracture had a worse survival rate than those without, according to Lee et al. [10]. Previously, two meta-analyses [11, 12] were conducted to clarify whether pathological fracture predicts poor prognosis in patients with osteosarcoma. Salunke et al. [12] found that the development of a pathological fracture was associated with a lower 5-year event-free survival (EFS) rate and possibly a higher local recurrence rate. Yang et al. [11] confirmed that pathological fracture in osteosarcoma was a prognostic marker for both overall survival (OS) and EFS but not for local recurrence. However, other studies have been reported that may significantly change this conclusion.

To provide clinical practice guidance and a framework for future research into this important question, we conducted the present meta-analysis with a systematic review of the literature and evaluated the association between pathological fracture and prognosis in patients with osteosarcoma.

## RESULTS

### Search process and characteristics of included studies

**Figure 1:**
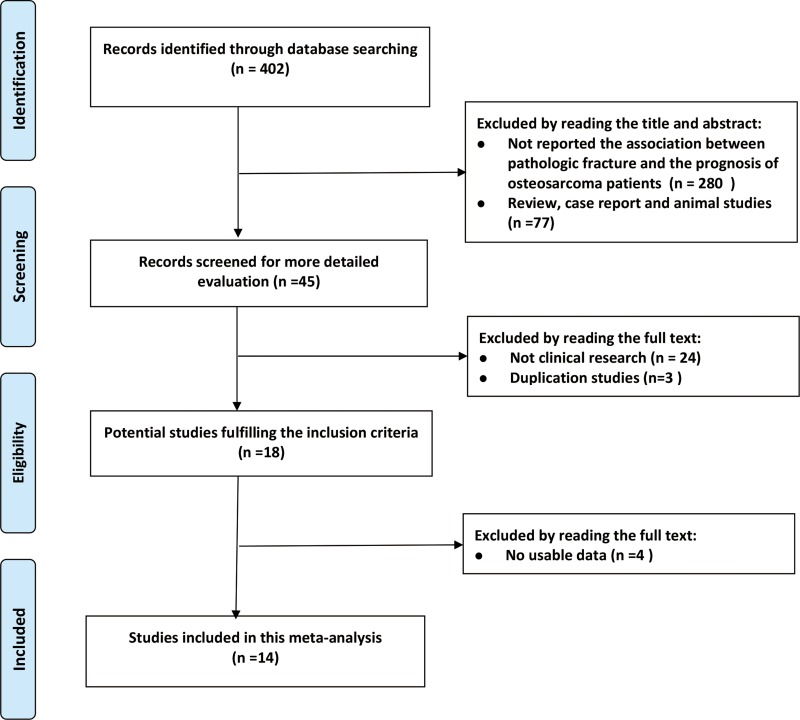
Flow chart showing the selection process for the included studies

The selection process for studies included in the present meta-analysis is shown in Figure 1. A total of 402 studies were retrieved from three databases: PubMed, Cochrane Library, and Web of Science. After screening of titles and abstracts, 280 studies were excluded because they did not report on the association between pathological fracture and prognosis of osteosarcoma patients. A further 77 studies were excluded because they were either reviews, case reports or animal studies. Upon detailed evaluation of the remaining 45 studies, we excluded 24 because they did not include clinical research and three were duplications studies of the same study population. Of the remaining 18 papers, four were eliminated because they had no usable data after full-text review. Eventually, 14 studies [7, 9, 10, 13–23] with 3910 patients were included in the present meta-analysis, including 541 pathological fracture patients and 3369 non-pathological fracture patients. All studies were published in the English language and were conducted between 1992 and 2016, with sample sizes ranging between 15 and 982 patients. Of these included studies, seven were performed in Asia, four in North America and three in Europe. Based on the results of Newcastle–Ottawa Scale (NOS) garding, all included studies were judged to be of high quality (Table 1).

**Table 1:** Newcastle–Ottawa scale for the assessment of the quality of included studies

Study/year	Selection				Outcome				Total score
	Case definition adequacy	Representativeness of case	Selection of controls	Definition of controls	Comparability of cases and controls	Ascertainment of exposure	Same method of ascertainment for cases and controls	Non-response rate	
Glasser et al./1992	*	*	*	*	*	*	*	*	8
Abudu et al./1996	*	*		*	*	*	*	*	7
Scully et al./1996	*	*	*	*	*	*	*	*	8
Scully et al./2002	*	*	*	**	*	*	*	*	9
Bacci et al./2003	*	*	*	*		*	*	*	7
Bramer et al./2007	*	*	*	**		*	*	*	7
Kim et al./2009	*	*	*	*	*	*	*	*	8
Cho et al./2010	*	*	*	*	*	*	*	*	8
Ferguson et al./2010	*	*	*	*		*	*	*	7
Xie et al./2012	*	*	*	*	*	*	*	*	8
Lee et al./2013	*	*		*	*	*	*	*	7
Zuo et al./2013	*	**	*	*	*	*	*	*	9
Deng et al./2015	*	*	**	*	*	*	*	*	9
Chung et al./2016	*	*	*	*	*	*	*	*	8

All of the included studies were retrospective cohort studies. Twelve reports were single-center, and two were multi-institutional. In the pathological fracture and non-pathological fracture groups, nine studies [9, 10, 14, 15, 17–19, 21, 22] reported local recurrence, four [9, 10, 14, 21] reported distant metastasis, three [9, 14, 15] reported 3-year OS, four [9, 14, 15, 20] reported 3-year EFS, six [9, 10, 15, 17, 21, 22] reported 5-year OS and seven [9, 13, 15–18, 20] reported 5-year EFS rates. Local recurrence data on amputation and limb salvage in pathological fracture patients was provided in 10 studies [7, 10, 14, 15, 17–20, 22, 23] (Table 2). Moreover, the treatments for patients and adjusted factors are shown in Table 3. Other factors, like age and gender, are shown in Supplementary Table 1.

**Table 2:** The characteristics of the selected clinical trials in this meta-analysis

Study/year	Country/Area	Study type	Age/year, mean (range)	Median Follow up time/month,median (range)	Enneking staging	Case/n (PF/non-PF)	LR/n (PF/non-PF)	Distant metastasis/n (PF/non-PF)	OS/% (PF/non-PF)		EFS/% (PF/non-PF)		Limb salvage/amputation in PF	
									3-year	5-year	3-year	5-year	Case/n	LR/n
Glasser et al./1992 [16]	USA	Retrospective cohort; single center	16 (3–63)	92 (36–156)	stage II	48/231	/	/	/	/	/	73.0/70.0	/	/
Abudu et al./1996 [23]	United Kingdom	Retrospective cohort; single center	18 (2–46)	55 (8–175)	stage IIB	/	/	/	/	/	/	/	27/13	5/0
Scully et al./1996 [7]	USA	Retrospective cohort; single center	18(11–68)	/	stage IIB	/	/	/	/	/	/	/	10/6	3/0
Scully et al./2002 [20]	USA	Retrospective cohort; multi-center	17 (2–69)	54 (6–152)	stage IIB	52/55	/	/	/	/	67.0/77.0	55.0/77.0	30/22	7/4
Bacci et al./2003 [17]	Italy	Retrospective cohort; single center	11 (3–20)	132 (36–240)	stage IIB	46/689	2/33	/	/	65.0/67.0	/	59.0/61.0	34/11	1/1
Bramer et al./2007 [19]	Netherlands	Retrospective cohort; single center	16 (4–57)	117 (7–252)	stage IIB	56/428	8/60	/	/	/	/	/	44/12	7/2
Kim et al./2009 [18]	Korea	Retrospective-cohort- and case-control; single center	/	43 (10–228)	AJCC stage II^a^	37/74	4/4	/	/	/	/	47.8/61.5	33/4	4/0
Cho et al./2010 [13]	Korea	Retrospective cohort; single center	19 (3–63)	84 (6–204)	AJCC stage II^a^	38/339	/	/	/	/	/	49.3/65.1	/	/
Ferguson et al./2010 [15]	Canada	Retrospective cohort; single center	30 (11–82)	/	/	31/201	2/18	/	52.0/78.0	52.0/68.0	44.0/64.0	44.0/60.0	19/12	2/0
Xie et al./2012 [9]	China	Retrospective cohort; single center	14 (6–30)	49 (9–102)	stage IIB	28/171	4/15	14/64	50.5/71.0	45.5/61.9	45.1/62.4	40/54.9	/	/
Lee et al./2013 [10]	China	Retrospective cohort; single center	13 (9–15)	/	/	5/10	1/2	3/1	/	40.0/80.0	/	/	2/3	1/1
Zuo et al./2013 [14]	China	Retrospective cohort; single center	23 (12–42)	35 (8–47)	stage IIB	15/50	4 /7	4 /16	66.7/75.3	/	53.3/66.5	/	10/5	3/1
Deng et al./2015 [22]	China	Retrospective cohort; multi-center	17 (4–75)	29 (1–220)	/	95/887	10/107	/	/	59.4/63.1	/	/	59/36	5/1
Chung et al./2016 [21]	Taiwan	Retrospective cohort; single center	/	/	stage IIB	34/234	8/51	17/75	/	37.0/50.0	/	/	/	/

a:AJCC, American Joint Committee on Cancer; PF: pathological fracture; LR: local recurrence; OS: overall survival; EFS: event-free survival;/:data not available.

**Table 3:** Treatments for patients and adjusted factors in included studies

Study/year	Treatments	Adjusted factors
Glasser et al./1992 [16]	Two hundred forty patients (86%) received preoperative chemotherapy, and all received postoperative chemotherapy. Definitive surgery consisted of amputation in 106 patients (38%), limb-sparing en bloc excision in 164 (59%), and excision with Van Nes rotationplasty in nine (3%).	Gender, age at diagnosis, location of fracture, ethnicity, clinical staging, treatment
Abudu et al./1996 [23]	All the patients were offered preoperative chemotherapy consisting of adriamycin and cisplatinum or adriamycin, cisplatinum and methotrexate according to the protocol of the European Osteosarcoma Intergroup. All had surgery after two to four cycles of chemotherapy except for one who had immediate amputation because of severe pain.	Age, location of fracture, gender, time of fracture, treatment
Scully et al./1996 [7]	Group I was treated nonoperatively with radiation or chemotherapy or both after the patients declined surgical resection; Group I1 had early amputation and subsequent chemotherapy; and Group I11 had neoadjuvant chemotherapy, limb salvage resection, followed by adjuvant chemotherapy.	Location of fracture, treatment
Scully et al./2002 [20]	Chemotherapeutic regimens varied with each institution and era of treatment. Chemotherapy protocols were the standard ones used at the time of tumor presentation.	Age at surgery, gender, year of surgery, anatomic location, tumor size on anteroposterior radiograph, tumor grade, type of resection, time of fracture, treatment, stabilization of fracture, fracture union, fracture displacement, tumor management
Bacci et al./2003 [17]	Preoperative chemotherapy was given.4–10 weeks, according to the protocol used. Postoperatively, chemotherapy was usually started within 7 days.	Gender, age, radiographic pattern, histology, serum alkaline phosphatase, location of fracture, surgical margins, tumor necrosis
Bramer et al./2007 [19]	All patients received standard treatment. This consisted of a pre-operative chemotherapy, followed by resection of the tumor and post-operative chemotherapy. For osteosarcoma chemotherapy was administered according to the protocol of the European Organisation for Research and Treatment of Cancer (EORTC)	Grade, gender, age, treatment
Kim et al./2009 [18]	Underwent standard therapy (neoadjuvant chemotherapy, definitive surgery, and adjuvant chemotherapy)	Age, gender, tumor diameter, tumor volume, location of fracture, radiograph, pathologic subtype, histologic response, final outcome
Cho et al./2010 [13]	All patients received standard therapy (neoadjuvant chemotherapy, definitive surgery, and adjuvant chemotherapy).	Age, gender, tumor volume, pattern on plain radiograph, pathologic subtype, operation type, tumor-volume ratio, histologic response
Ferguson et al./2010 [15]	The chemotherapy regimen utilized was individualized in each case but patients under 40 typically received adriamycin, cisplatin, and methotrexate, whereas those over 40 only received adriamycin and cisplatin.	Gender, age, timing of fracture, fracture displaced, fracture management
Xie et al./2012 [9]	All the patients with pathologic fracture were immobilized using plasters, braces, or other orthopedic appliances. All patients underwent 1–2 cycles of neo-adjuvant chemotherapy and 4–6 cycles of adjuvant chemotherapy.	Age, gender, location, size, histological subtype, ALP levels, radiographic features
Lee et al./2013 [10]	All patients in both index group received neoadjuvant chemotherapy prior to operation. All pathological fractures in the index group healed before operation.	Age, treatment, size
Zuo et al./2013 [14]	Each of the 15 patients of the fracture group was immobilized by standard brace or plaster cast. Patients were then followed for a minimum of 4 preoperative adjuvant chemotherapy cycles, according to the National Comprehensive Cancer Network (NCCN).	Gender, age, site, stage, surgery, displacement, tumor N stage, subtype, FP time
Deng et al./2015 [22]	All patients with pathological fracture were immobilized immediately after fracture by skeletal traction or cast. No internal fixation was employed. Neoadjuvant chemotherapy was given according to the respective hospital protocol, and immobilization was continued during this period.	Age, gender, anatomicallocation, treatment
Chung et al./2016 [21]	The pre-operative neoadjuvant chemotherapy regimen was standardized after 2003. Adjuvant chemotherapy following surgery was provided according to the guidelines of National Comprehensive Cancer Network (NCCN) for bone cancer. After chemotherapy and reassessment, all patients received the definite tumor surgery based on their responses to chemotherapy, location and extension of tumor, and patient age, to achieve wide surgical margins as much as possible.	Gender, age, stage of tumor, tumor size, tumor location, lung metastasis, necrosis rate, local recurrence, duration to recurrence, follow up duration, status until last follow up

### Meta-analysis of the prognostic value of pathological fracture in osteosarcoma

**Figure 2:**
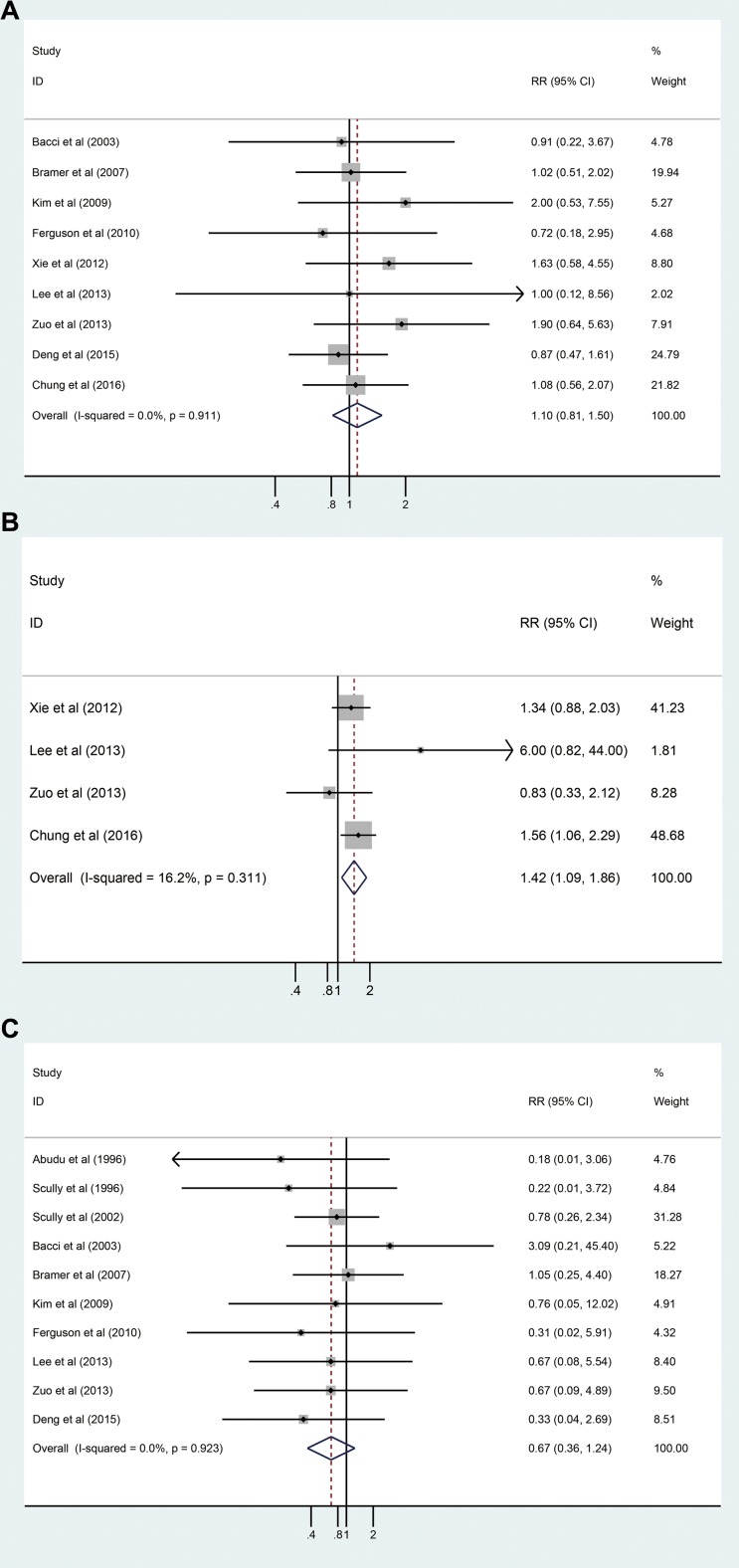
(**A**) Forest plot of the RR_adj_ for local recurrences analysis; (**B**) Forest plot of the RR_adj_ for distant metastasis analysis; (**C**) Forest plot of the RR_adj_ for local recurrence between limb slavery and amputation group analysis.

**Figure 3:**
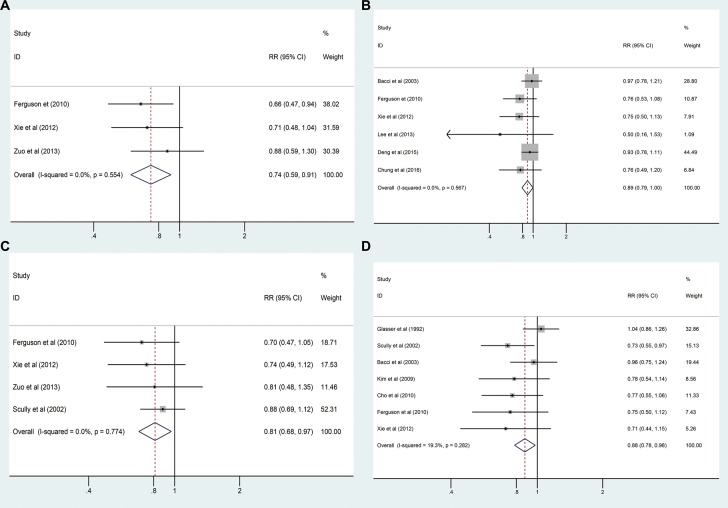
(**A**) Forest plot of the RRadj for 3-year OS analysis; (**B**) Forest plot of the RR for 5-year OS analysis; (**C**) Forest plot of the RRadj for 3-year EFS analysis; (**D**) Forest plot of the RRadj for 5-year EFS analysis.

As shown in Table 4, local recurrence data from nine studies with 3091 patients were combined for analysis, and no statistically significant difference was detected between the pathological fracture and non-pathological fracture groups (RR_adj_ = 1.102, 95% CI: 0.813–1.495, *P* = 0.531, Figure 2A). No significant heterogeneity between these studies (*I*^2^ = 0.0%, *P* = 0.911) was observed. We also combined distant metastasis of the pathological fracture and non-pathological fracture groups, and a statistically significant difference was found between the two groups (RR_adj_ = 1.424, 95% CI: 1.089–1.862, *P* = 0.01, Figure 2B). No significant heterogeneity was detected (*I*^2^ = 16.2%, *P* = 0.311). The following were identified for survival rates: 3-year OS (RR_adj_ = 0.736, 95% CI: 0.593–0.912, *P* = 0.554, *I*^2^ = 0.0%, Figure 3A); 5-year OS (RR_adj_ = 0.889, 95% CI: 0.791–0.999, *P* = 0.567, *I*^2^ = 0.0%, Figure 3B); 3-year EFS (RR_adj_ = 0.812, 95% CI: 0.682–0.966, *P* = 0.774, *I*^2^ = 0.0%, Figure 3C); and 5-year EFS (RR_adj_ = 0.876, 95% CI: 0.785–0.978, *P* = 0.282, *I*^2^ = 19.3%, Figure 3D). The results showed that osteosarcoma patients with pathological fracture had poorer survival rates compared to those without pathological fracture (Table 4).

**Table 4:** Results in the overall analysis

Group	Study	Case (*n*)	RR/RR (adjusted)	95% CI/95% CI (adjusted)	P/P (adjusted)	Heterogeneity			P (publication bias)
						P/P (adjusted)	I^2^ (%)/I^2^ (%) (adjusted)	Statistical model	
LR^a^	9	3091	1.076/1.102	0.794–1.459/0.813–1.495	0.636/0.531	0.909/0.911	0/0	Fixed-effects model	0.409
Distant metastasis	4	547	1.417/1.424	1.082–1.855/1.089–1.862	0.011/0.01	0.311/0.311	16.2/16.2	Fixed-effects model	0.745
3-year OS	3	496	0.718/0.736	0.577–0.894/0.593–0.912	0.003/0.005	0.539/0.554	0/0	Fixed-effects model	0.402
5-year OS	6	2431	0.87/0.889	0.772–0.980/0.791–0.999	0.022/0.049	0.55/0.567	0/0	Fixed-effects model	0.01
3-year EFS	4	603	0.787/0.812	0.655–0.945/0.682–0.966	0.01/0.018	0.741/0.774	0/0	Fixed-effects model	0.241
5-year EFS	7	2040	0.847/0.876	0.755–0.950/0.785–0.978	0.004/0.019	0.252/0.282	23.2/19.3	Fixed-effects model	0.017
LR^b^	10	392	0.594/0.673	0.326–1.084/0.364–1.244	0.09/0.206	0.914/0.923	0/0	Fixed-effects model	0.259

LR: local recurrence; a: LR based on pathological fracture and non-pathological fracture patients; b: LR based on pathological fracture patients with Limb salvage or amputation; OS: overall survival; EFS: event-free survival.

### Meta-analysis of local recurrence between limb slavery and amputation group on pathologic fracture patients

Local recurrence data from amputation and limb salvage groups in 392 pathological fracture patients from 10 studies were also combined for analysis. The pooled estimate of RR_adj_ was 0.673 (95% CI: 0.364–1.244, Figure 2C), and no significant heterogeneity was detected (*I*^2^ = 0.0%, *P* = 0.923) (Table 4).

### Subgroup analysis based on tumor stage IIB

In subgroup analysis based on tumor stage IIB, we performed subgroup analysis if there were 2 or more studies reporting on a particular characteristic [24]. The results showed that osteosarcoma patients with pathological fracture were more likely to have distant metastasis and poorer 3-year EFS, and 5-year EFS compared to those without pathological fracture (Table 5).

**Table 5:** Results in the subgroup analysis based on tumor stage IIB

Group	Study	Case (n)	RR/RR (adjusted)	95% CI/95% CI (adjusted)	P/P (adjusted)	Heterogeneity		
						P/P (adjusted)	I^2^(%)/I^2^(%) (adjusted)	Statistical model
LR^a^	5	1751	1.169/1.19	0.797-1.714/0.811-1.746	0.424/0.375	0.824/0.825	0/0	Fixed-effects model
Distant metastasis	3	532	1.348/1.386	1.026-1.772/1.058-1.817	0.032/0.018	0.454/0.463	0/0	Fixed-effects model
3-year OS	2	264	0.765/0.786	0.577-1.013/0.598-1.033	0.062/0.084	0.424/0.44	0/0	Fixed-effects model
5-year OS	3	1202	0.863/0.893	0.718-1.038/0.749-1.066	0.117/0.211	0.389/0.416	0/0	Fixed-effects model
3-year EFS	3	404	0.801/0.827	0.655-0.981/0.683-1.002	0.032/0.052	0.604/0.64	0/0	Fixed-effects model
5-year EFS	3	1041	0.829/0.833	0.693-0.991/0.700-0.992	0.039/0.041	0.284/0.284	20.6/20.6	Fixed-effects model
LR^b^	6	224	0.667/0.76	0.334-1.332/0.372-1.553	0.251/0.452	0.685/0.702	0/0	Fixed-effects model

LR: local recurrence; ^a^: LR based on pathological fracture and non-pathological fracture patients; ^b^: LR based on pathological fracture patients with Limb salvage or amputation; OS: overall survival; EFS: event-free survival.

### Publication bias and sensitivity analysis

**Figure 4:**
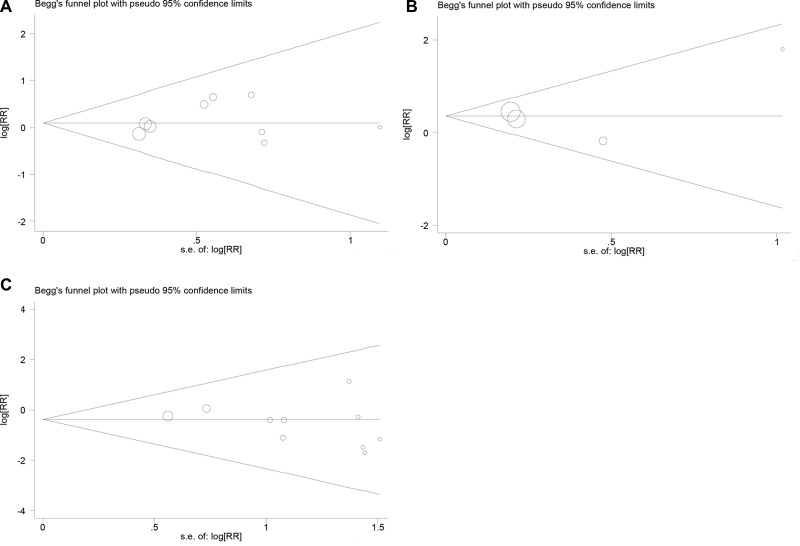
(**A**) Funnel plot for local recurrences analysis; (**B**) Funnel plot for distant metastasis analysis; (**C**) Funnel plot for local recurrence between limb slavery and amputation group analysis.

**Figure 5:**
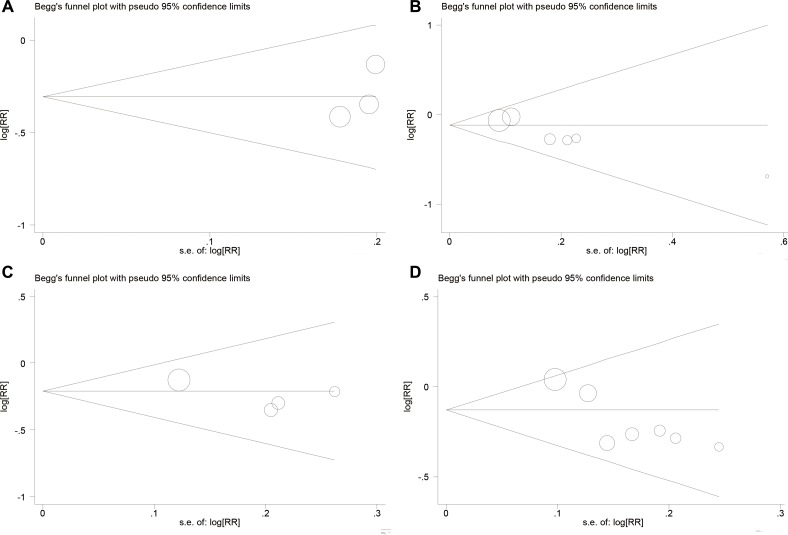
(**A**) Funnel plot for 3-year OS analysis; (**B**) Funnel plot for 5-year OS analysis; (**C**) Funnel plot for 3-year EFS analysis; (**D**) Funnel plot for 5-year EFS analysis.

**Figure 6:**
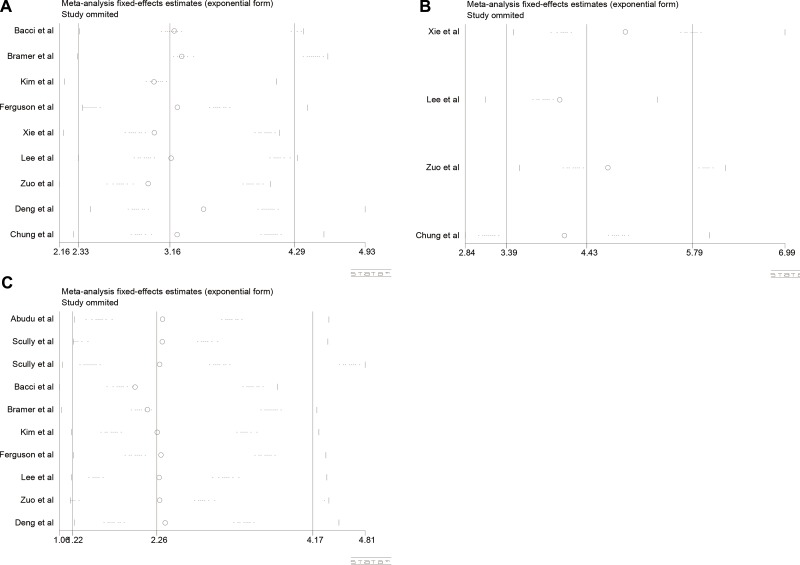
(**A**) Sensitivity analysis for local recurrences analysis; (**B**) Sensitivity analysis for distant metastasis analysis; (**C**) Sensitivity analysis for local recurrence between limb slavery and amputation group analysis.

**Figure 7:**
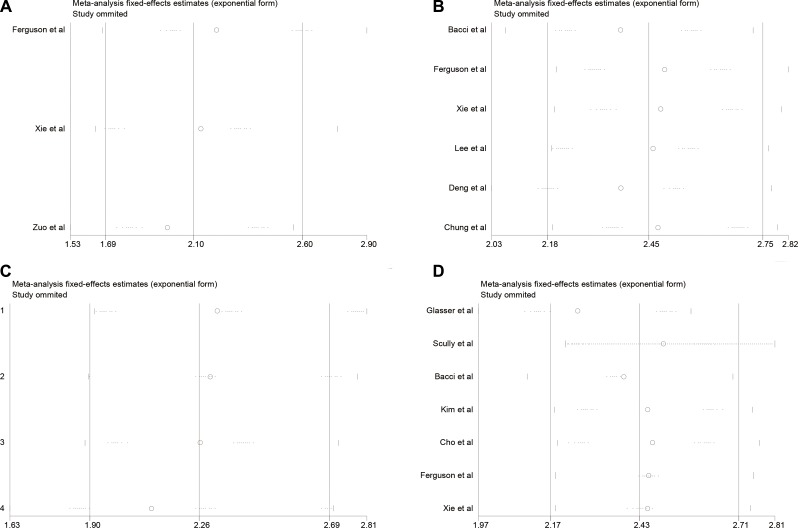
(**A**) Sensitivity analysis for 3-year OS analysis; (**B**) Sensitivity analysis for 5-year OS analysis; (**C**) Sensitivity analysis for 3-year EFS analysis; (**D**) Sensitivity analysis for 5-year EFS analysis.

In this meta-analysis, we used Begg's funnel plot to test publication bias. As shown in Table 4, Figure 4, and Figure 5, the results suggested no significant publication bias except for 5-year OS (*P* = 0.010, Figure 5B) and 5-year EFS (*P* = 0.017, Figure 5D). Sensitivity analysis was conducted by omitting studies one by one and analyzing the remaining studies. As shown in Figure 6 and Figure 7, the results were not substantially changed, highlighting the reliability and stability of our results.

## DISCUSSION

The 5-year OS for osteosarcoma reached 65%–70% due to the development of high-dose chemotherapy and surgical progress [2]. However, in the past 20 years, there were no further improvements in osteosarcoma survival. To explain this lack of progress, many studies were performed to investigate factors effecting the progression and metastasis of osteosarcoma, such as levels of C-reactive protein and expression of hypoxia-inducible factor-1 alpha [25, 26]. There are several studies which have assessed the association between pathological fracture and prognosis in patients with osteosarcoma; however, the results were inconclusive because of the limited sample of single-study. Thus, we used a pooled meta-analysis of current published studies to further evaluate the association between pathological fracture and prognosis in patients with osteosarcoma.

Previously, Salunke et al. [12] performed a meta-analysis of eight articles (303 patients with pathological fractures and 1410 without fractures). Their findings showed that pathological fracture is negative prognostic predictor in osteosarcoma and is related to reduced 5-year EFS and possibly a higher rate of local recurrence. Yang et al. [11] analyzed a total of 1,677 subjects. Their results demonstrated that osteosarcoma patients with pathological fracture have worse survival outcomes, including OS and disease-free survival (DFS), and furthermore that pathological fracture may be a poor indicator of survival in osteosarcoma patients. Compared with the above two meta-analyses, we included 14 articles with 3910 patients, nearly double the sample size, which helps to strengthen statistical power. Moreover, distant metastasis was analyzed in our meta-analysis and in addition, subgroup analysis based on tumor stage IIB was conducted. We also performed sensitivity analysis, which was not conducted in the above meta-analyses.

We evaluated the association between pathological fracture and prognosis in osteosarcoma in 541 pathological fracture patients and 3369 non-pathological fracture patients. We found no difference in local recurrence between patients with or without a fracture. Moreover, there was no difference in the rate of local recurrence after limb salvage in patients with pathological fracture. Furthermore, the patients with pathological fracture had higher risk of distant metastasis, and poorer 3-year OS, 5-year OS, 3-year EFS, and 5-year EFS. In subgroup analysis based on tumor stage IIB, the results showed that osteosarcoma patients with pathological fracture were more likely to have distant metastasis and poorer 3-year EFS and 5-year EFS compared to those without pathological fracture. In fact, fracture was found to be an independent prognostic indicator of worse survival; however, there was no difference in local recurrence. This indicated that it is probably not the spreading of tumor cells in the fracture hematoma that contributes to worse prognosis.

Interestingly, the rate of distant metastasis in patients with pathological fracture is higher than patients without pathological fracture. Lee et al. [10] postulated that if the osteosarcoma is more aggressive with more cortical and marrow infiltration, the chance of pathological fracture and vascular invasion would increase. Thus, invasion may destroy the architecture of the bone and increase the chance of pathological fracture and the degree of vascular invasion. Moreover, a much higher proportion of patients with fractures have lung metastases than those without (50% vs. 32%, respectively) [21].

Notably, limb salvage did not greatly increase the risk for local recurrence compared with amputation in our analysis. Moreover, we are also interested in the correlation between the location of pathological fracture and local recurrence; however, a detailed analysis was not conducted. Previous studies [7, 14] have indicated that local recurrences occurred in the proximal humerus, the distal femur, and the proximal femur. Furthermore, in patients with a pathological fracture, stabilization of fracture, such as open reduction and internal fixation or closed immobilization, has been shown not to modify local recurrence [20].

It is well established that amputation can reduce the quality of life for patients. A large proportion of amputees (60–80%) experience the phenomenon of phantom limbs [27, 28]. They feel pain even though that body parts is no longer there. These limbs can itch, burn, cause pain, feel locked in or trapped and feel as if they are moving [29]. In contrast, limb salvage is performed as an alternative to amputation. A study indicated that patients who underwent a limb salvage procedure at least 10 years before had better quality of life [30]. Thus, we advocate for the use of limb salvage in selected patients.

Whether pathological fracture affects long-term survival seems more controversial. Based on Xie et al.'s study [9], the OS rate at 3 years was 50.5% in the fracture group and 71.0% in the group of patients without a pathological fracture. Continuous EFS rate at 3 years was 45.1% in the fracture group and 62.4% in the group of patients without a pathological fracture. Moreover, early in 2003, Bacci et al. [17] showed that the 5-year EFS was 59% in the pathological fracture group versus 61% in the non-pathological. Moreover, the 5-year OS was 65% in the pathological fracture group and 67% in the non-pathological. Our study demonstrated that pathological fracture affects long-term survival. However, the clear mechanism is still unknown and more studies are necessary.

Notably, the results show publication bias in relation to 5-year OS and 5-year EFS. However, the results of sensitivity analysis were not significantly changed, highlighting the reliability and stability of our results. Importantly, there are several limitations with the present meta-analysis. Firstly, our study only considered the impact of pathological fracture; other established prognostic factors, such as tumor stage, size, and chemotherapy response, were not considered due to limited information. Secondly, relevant studies in other languages were excluded, because the included publications were mainly written in English. Thirdly, only published studies with available data were analyzed in our study, so therefore unpublished data might influence the results. In addition, some studies [16, 23] old and the results may differ considerably from studies conducted more recently. Furthermore, variability in treatment regimens is a consistent bias found in meta-analyses. For example, neoadjuvant chemotherapy is important for long-term survival in patients with osteosarcoma; however, some studies neither used this method nor used neoadjuvant chemotherapy based on the respective hospital protocol. Lastly, although we included more studies than previous meta-analyses, the number of publications included was still limited.

In conclusion, our meta-analysis indicated that there were no differences in local recurrence and local recurrence after limb salvage between patients with or without a pathological fracture. Furthermore, the patients with pathological fracture had higher risk for distant metastasis, and poor 3-year OS, 5-year OS, 3-year EFS, and 5-year EFS. Considering the aforementioned limitations, we think future large-scale studies should be performed to confirm our conclusions.

## MATERIALS AND METHODS

### Literature search

We searched three databases: PubMed, Web of Science, and Embase up to May 15, 2017 without any language restrictions. The keywords were: (pathologic fracture OR pathological fracture OR spontaneous fracture) and (osteosarcoma OR osteosarcoma tumor OR osteogenic sarcomas) and (amputation OR limb salvage OR prognosis). The search results were supplemented by screening references of the original articles and systematic reviews.

### Inclusion and exclusion criteria

Studies were included if they met the following criteria: clinical human studies; all patients were diagnosed with osteosarcoma with or without pathological fracture; the association between pathological fracture and the prognosis of osteosarcoma patients was examined and/or treatment for pathological fracture patients including limb salvage and amputation and the prognosis was compared; the study had available data. The exclusion criteria were: abstracts or reviews; studies reporting duplicate data; no usable data; non clinical and/or non-human studies.

### Data extraction

Two authors reviewed each eligible article and extracted the data independently. All of the differences and contradictions were resolved by a third investigator. The major information from each enrolled study was extracted: first author; year of publication; country; study type; mean age and range; median follow-up time and range; Enneking staging [31] of patients; the number of pathological fracture and non-pathological fracture groups; the prognosis measures including local recurrence, distant metastasis, 3-year OS, 5-year OS, 3-year EFS and 5-year EFS in the pathological fracture and non-pathological fracture groups; the number of amputation and limb salvage groups among pathological fracture patients, as well as the local recurrence rate between the two groups.

### Quality assessment

The NOS for cohort studies was used to assess the quality of included studies [32]. Two authors independently conducted the quality assessments. NOS is comprised of three parameters for quality: selection, comparability, and outcome assessment. In the selection and outcome categories, a quality research item received one star, and a comparable category could receive at most two stars. Furthermore, each study received a total score between 0 and 9, with a NOS score of 7 or above considered as high quality and a NOS score of 3 or below considered as low quality.

### Statistical analysis

The RR with 95% CI was used to compare binary data between the case and control groups, including local recurrence, distant metastasis, 3-year OS, 5-year OS, 3-year EFS and 5-year EFS. We applied the I^2^-statistic to calculate heterogeneity among the studies (I^2^ > 50% implies significant heterogeneity) and the random-effects model was chosen, otherwise a fixed-effects model was used [33]. For crude data analysis, we used the number of people with or without pathological fracture in the case and control group. For the analysis of the adjusted data, we extracted the RR with 95% CI that had been adjusted for various potential confounders [34, 35]. Publication bias was estimated by Begger's funnel plot in overall analysis. Sensitivity analysis was conducted to evaluate whether modification of our inclusion criteria influenced the final results in overall analysis. *P* values < 0.05 were considered statistically significant [36]. All statistical analyses were conducted using STATA software (version12.0, STATA Corp., College Station, TX, USA).

## SUPPLEMENTARY TABLE



## Abbreviations

NOS: Newcastle-Ottawa scale
OS: overall survival
EFS: event free survival
RR: relative risk
CI: confidence intervals
