# Supplementary material for: The effect of pathological fractures on the prognosis of patients with osteosarcoma: a meta-analysis of 14 studies
Source: Oncotarget. 2017 Aug 21;8(42):73037–49. doi: 10.18632/oncotarget.20375 (PMC5641190; doi:10.18632/oncotarget.20375)
Supplement: Supplementary file 1 [file oncotarget-08-73037-s001.pdf]

The effect of pathological fractures on the prognosis of patients with osteosarcoma: a meta-analysis of 14 studies

SUPPLEMENTARY MATERIALS

Supplementary Table 1: Some other factors in included studies

Study/year	Group	Gender		Location of fracture				Time of fracture			Type of resection			
		Male	Female	Femur	Humerus	Tibia	Other	At diagnosis	During treatment	After biopsy	Marginal	Wide	Radical	Intralesional
Glasser et al./1992	PF	/	/	/	/	/	/	/	/	/	/	/	/	/
	non-PF	/	/	/	/	/	/	/	/	/	/	/	/	/
Abudu et al./1996	PF	26	14	25	12	3	0	25	15	/	/	/	/	/
	non-PF	/	/	/	/	/	/	/	/	/	/	/	/	/
Scully et al./1996	PF	/	/	/	/	/	/	/	/	/	/	/	/	/
	non-PF	/	/	/	/	/	/	/	/	/	/	/	/	/
Scully et al./2002	PF	28	24	34	13	4	1	/	/	/	2	38	12	0
	non-PF	30	25	36	13	4	2	/	/	/	6	42	6	0
Bacci et al./2003	PF	24	22	22	16	5	3	/	/	/	3	34	6	3
	non-PF	/	/	/	/	/	/	/	/	/	50	593	29	12
Bramer et al./2007	PF	36	20	/	/	/	/	/	/	/	/	/	/	/
	non-PF	254	174	/	/	/	/	/	/	/	/	/	/	/
Kim et al./2009	PF	26	11	16	16	1	4	/	/	/	8	25	4	0
	non-PF	47	27	32	32	2	8	/	/	/	4	67	3	0
Cho et al./2010	PF	/	/	/	/	/	/	/	/	/	/	/	/	/
	non-PF	/	/	/	/	/	/	/	/	/	/	/	/	/
Ferguson et al./2010	PF	14	17	21	7	3	0	20	6	5	/	/	/	/
	non-PF	/	/	/	/	/	/	/	/	/	/	/	/	/
Xie et al./2012	PF	20	8	17	7	3	1	/	/	/	/	/	/	/
	non-PF	107	64	90	14	57	10	/	/	/	/	/	/	/
Lee et al./2013	PF	/	/	/	/	/	/	/	/	/	/	/	/	/
	non-PF	/	/	/	/	/	/	/	/	/	/	/	/	/
Zuo et al./2013	PF	8	7	8	5	2	0	/	/	/	/	/	/	/
	non-PF	/	/	/	/	/	/	/	/	/	/	/	/	/
Deng et al./2015	PF	55	40	68	20	5	2	/	/	/	/	/	/	/
	non-PF	491	396	526	88	243	30	/	/	/	/	/	/	/
Chung et al./2016	PF	19	15	18	10	3	3	/	/	/	/	/	/	/
	non-PF	141	93	94	16	49	75	/	/	/	/	/	/	/

PF: pathological fracture; non-PF: non-pathologic fracture; /: not available.
